# Stress Responsiveness of the Hypothalamic–Pituitary–Adrenal Axis: Age-Related Features of the Vasopressinergic Regulation

**DOI:** 10.3389/fendo.2013.00026

**Published:** 2013-03-12

**Authors:** Nadezhda D. Goncharova

**Affiliations:** ^1^Research Institute of Medical Primatology of Russian Academy of Medical SciencesSochi, Russia; ^2^Sochi State UniversitySochi, Russia

**Keywords:** hypothalamic–pituitary–adrenal axis, vasopressin, stress, aging, V1b receptors

## Abstract

The hypothalamic–pituitary–adrenal (HPA) axis plays a key role in adaptation to environmental stresses. Parvicellular neurons of the hypothalamic paraventricular nucleus secrete corticotrophin releasing hormone (CRH) and arginine vasopressin (AVP) into pituitary portal system; CRH and AVP stimulate adrenocorticotropic hormone (ACTH) release through specific G-protein-coupled membrane receptors on pituitary corticotrophs, CRHR1 for CRH and V1b for AVP; the adrenal gland cortex secretes glucocorticoids in response to ACTH. The glucocorticoids activate specific receptors in brain and peripheral tissues thereby triggering the necessary metabolic, immune, neuromodulatory, and behavioral changes to resist stress. While importance of CRH, as a key hypothalamic factor of HPA axis regulation in basal and stress conditions in most species, is generally recognized, role of AVP remains to be clarified. This review focuses on the role of AVP in the regulation of stress responsiveness of the HPA axis with emphasis on the effects of aging on vasopressinergic regulation of HPA axis stress responsiveness. Under most of the known stressors, AVP is necessary for acute ACTH secretion but in a context-specific manner. The current data on the AVP role in regulation of HPA responsiveness to chronic stress in adulthood are rather contradictory. The importance of the vasopressinergic regulation of the HPA stress responsiveness is greatest during fetal development, in neonatal period, and in the lactating adult. Aging associated with increased variability in several parameters of HPA function including basal state, responsiveness to stressors, and special testing. Reports on the possible role of the AVP/V1b receptor system in the increase of HPA axis hyperactivity with aging are contradictory and requires further research. Many contradictory results may be due to age and species differences in the HPA function of rodents and primates.

## Introduction

The hypothalamic–pituitary–adrenal (HPA) axis is a key adaptive neuroendocrine system. Regulation of glucocorticoid secretion through adrenocorticotropic hormone (ACTH) is critical to life and essential to maintain the mammalian response to stressor (Pedersen et al., 2001; McEwen, 2007; Lupien et al., 2009). The HPA axis consists of the nucleus paraventricularis hypothalami (PVN), which secretes corticotrophin releasing hormone (CRH) and arginine vasopressin (AVP); the pituitary gland that is sensitive to CRH and AVP, which stimulate ACTH release; the adrenal gland cortex, which secretes the glucocorticoids, mainly cortisol in humans and non-human primates, and corticosterone in rodents, and dehydroepiandrosterone (DHEA) in humans and non-human primates in response to interaction with ACTH. DHEA and its sulfate DHEAS are important regulators of glucocorticoid activity (Yen and Laughlin, 1998; Maninger et al., 2009). The glucocorticoids act on specific receptors present in most peripheral tissues and the brain and trigger the metabolic, immune, neuromodulatory, and behavioral changes needed to cope with the impact of the stressors (Tuckermann et al., 2005; McEwen, 2007; Vegiopoulos and Herzig, 2007). In turn, the glucocorticoids act through the pituitary and limbic structures, especially the hippocampus, in a negative feedback loop to regulate the activity of CRH-producing neurons in the PVN and, thus the entire the HPA axis. The adaptive influence of the HPA axis under stress is realized not only through ACTH and the corticosteroids but also through the hypothalamic neuropeptides CRH and AVP, which are released in the brain, where they are responsible for behavioral and autonomic responses to stress (Bale and Vale, 2004; Roper et al., 2011).

In resting conditions, activity of the HPA axis shows circadian and ultradian changes with pulsatile glucocorticoid secretion that is greater in amplitude during the phase of wakefulness. This leads to higher average levels of glucocorticoids during the day in humans and most other primates and to higher activity of the HPA axis during the night in rodents (Goncharova et al., 2002; Kalsbeek et al., 2010; Russell et al., 2010; Walker et al., 2010). Stress responsiveness of the pituitary–adrenal axis in humans and non-human primates also shows the circadian rhythms with higher stress response in the afternoon and evening, and lower in the morning (Goncharova, 2006; Goncharova et al., 2006a; De Weerth et al., 2007). Rhythmicity in the HPA axis is essential for the normal functioning of the brain and other glucocorticoid responsive organs (Van Cauter et al., 1997; Lupien et al., 2002; Yoshimura et al., 2007; Goncharova et al., 2008a,b).

While the acute stress activation of the HPA axis is critical for life, chronic exposure to stressors leads to its excessive stimulation and hypercortisolemia. Hypercortisolemia plays a pathophysiological role in the development of a variety of stress-related diseases: psychiatric, reproductive, immune, metabolic, and others. It is a major factor in aging and age-related pathology (Chrousos, 2000; de Kloet et al., 2005, 2008; Perez-Neri et al., 2008). While CRH is widely recognized as a major hypothalamic factor controlling the HPA axis activity in resting conditions and under stress, the role of AVP in regulation of the HPA axis remains to be studied. Almost all current data on the role of AVP in regulation of the HPA axis were obtained in experiments on adult rodents. In the available literature, there are no review articles devoted to the role of AVP in the regulation of HPA axis stress responsiveness in aged subjects. In any event, aging is generally characterized by hyperactivation of the HPA axis, breakdown of the circadian rhythm, progressive decline in DHEA and DHEAS production, and high incidence of stress-dependent diseases, including depression, in which AVP production and the corticosteroids are of great importance (Yen and Laughlin, 1998; de Winter et al., 2003; Dinan and Scott, 2005; Kondratova and Kondratov, 2012). This review will focus on the evaluation of the role of AVP in the regulation of stress responsiveness of the HPA axis with emphasis on HPA axis vasopressinergic regulation during healthy aging in rodents as well as in humans and non-human primates.

## Mechanisms of HPA Axis Regulation

### Corticotrophin releasing hormone

Corticotrophin releasing hormone is the main physiological regulator of the HPA axis in basal conditions and in response to most acute stressors (Jacobson et al., 2000; Bale and Vale, 2004; Aguilera, 2011). CRH is a peptide consisting of 41 amino acid residues, which was discovered and sequenced by Vale et al. (1983). It is produced in the medial parvicellular neurons of the PVN, which project to the outer zone of the median eminence. There it is released into the portal system of the pituitary, which reaches the anterior lobe. CRH stimulates ACTH secretion through activation of type 1 CRH receptors (CRHR1) in the pituitary corticotrophs. CRHR1 belongs to the CRH family of G-protein-coupled membrane receptors (GPCRs) (Bale and Vale, 2004; Zorrilla and Koob, 2010).

The collection of CRH-producing neurons (CRH neurons) of the PVN is a key center of the central nervous system, integrating the neuroendocrine effects of stress, and a key part of the HPA axis. On the one hand, the CRH neuron is under the regulatory influence of numerous afferent nerve pathways that carry information about the stressor. In addition, it is regulated by glucocorticoids and it is the central link of the autoregulation mechanism in the HPA axis.

#### Afferent regulation

The CRH neuron receives projections from ascending catecholaminergic pathways including noradrenergic projections from the A2 noradrenergic cell group within the nucleus of the solitary tract and the locus ceruleus. It also receives input from areas of the limbic system, notably the bed nucleus of the stria terminalis, the hippocampus, and the amygdala (Herman et al., 1996; Dinan and Scott, 2005). Indirect pathways from the sensory systems in the forebrain through the amygdala and bed nucleus of the stria terminalis may stimulate CRH neurons by activating stimulatory (glutamatergic) or repressing inhibitory (γ-aminobutyric acid – GABA-ergic) interneurons (Herman et al., 1996; Arvat et al., 2002; Aguilera, 2011). There is also significant evidence in relation to the stimulatory effect of the cholinergic system on CRH secretion implemented through direct and indirect projections from both the basal forebrain and the mesopontine tegmentum (Rhodes and Rubin, 1999; Rubin and Rhodes, 2002).

#### Glucocorticoid feedback mechanism

Feedback mechanism plays an essential role in the regulation of CRH production and in limiting the stress response. The central sensors of feedback to the HPA axis are two corticosteroid receptors, the high-affinity mineralocorticoid receptor (MR) and the low-affinity glucocorticoid receptors (GR), which are expressed in the brain and on the corticotrophs of the pituitary (Ratka et al., 1989). GRs are distributed throughout the brain, but mostly in hypothalamic neurons and corticotrophs (Jacobson and Sapolsky, 1991; Funder, 1997), whereas MRs are present in the hypothalamus and in the greatest numbers in the hippocampus (Ratka et al., 1989; Funder, 1997). A significant role of direct inhibition of transcription of pro-opiomelanocortin (POMC) and ACTH secretion by glucocorticoids in the regulation of the HPA axis by a feedback mechanism has been previously demonstrated (Autelitano et al., 1989; Dallman et al., 1994). Development of MR antagonists has enabled demonstration of the important role of glucocorticoid feedback at the suprapituitary level, probably from the hippocampus in regulation of the HPA axis both in animals and humans (Wellhoener et al., 2004; Giordano et al., 2005). The presence of GRs in CRH neurons (Liposits et al., 1987), as well as a demonstrated inhibitory effect of intra-PVN glucocorticoid injection on CRH mRNA, and the reduction in CRH promoter activity in reporter gene assays following incubation with glucocorticoids, give reason to believe that a direct inhibitory effect of glucocorticoids on CRH transcription underlies the central mechanisms of feedback regulation of CRH transcription and desensitization of the HPA axis to stress responses (Kovacs et al., 1986; Sawchenko, 1987; Harbuz and Lightman, 1989; Makino et al., 1995; Fenoglio et al., 2004; Aguilera, 2011).

### Arginine vasopressin

#### Distribution of AVP

While it is generally accepted that CRH is a critical coordinator of HPA axis function in resting conditions and in response to stress (Aguilera, 1998, 2011; Jacobson et al., 2000; Bale and Vale, 2004), the role of AVP in the regulation of the HPA axis remains a subject of active research. Following its identification in 1954, AVP, a nonapeptide, was considered the principal factor in the regulation of ACTH secretion until the subsequent identification of CRH, which led to the replacement of AVP with CRH as the principle regulator of the HPA axis. Nevertheless, AVP is still considered as an important regulator of the HPA axis (Antoni, 1993; Herman, 1995; Volpi et al., 2004; Aguilera, 2011). In addition, AVP is seen as a hormonal regulator of water homeostasis and has significant effects on vascular tone (Knepper, 1997; Koshimizu et al., 2006). AVP is also believed to regulate circadian rhythms (Kalsbeek et al., 2010; Kondratova and Kondratov, 2012) and numerous central functions such as synaptic transmission, control of body temperature, memory, and behavior (Barberis and Tribollet, 1996; Wersinger et al., 2002).

Knowledge of the functional anatomy, physiology, and pathophysiology of AVP in the regulation of the HPA axis is mainly based on studies carried out on rodents. There are two major vasopressinergic systems in the brain. The first system consists of hypothalamic magnocellular neurons in the PVN and supraoptic nucleus, which project to the neurohypophysis and deliver AVP and oxytocin to the peripheral circulation; it is largely responsible for the peripheral actions of AVP that maintain water homeostasis and blood pressure (Knepper, 1997; Koshimizu et al., 2006). The second system is represented by the CRH-producing parvicellular neurons of the PVN, which also synthesizes AVP. AVP produced by the latter neuron is secreted into the pituitary portal circulation from axon terminals in the external zone of the median eminence; they are involved in regulation of the HPA axis (Antoni, 1993). It has been shown in non-stressed rats that 50% of CRH neurosecretory cells coexpress AVP (Whitnall, 1987). In humans, there is evidence that all CRH neurons in the hypothalamus may contain AVP (Mouri et al., 1993; Dinan and Scott, 2005). In primates, including humans, large numbers of immunoreactive AVP neurons have been demonstrated in the limbic system, notably the bed nucleus of the stria terminalis, the hippocampus and the amygdala, and also in the pituitary intermediate lobe, the granular layers of cerebellar cortex, and the dentate gyrus. Lower levels are found in the medial habenula, adenohypophysis, area postrema, pineal body, subfornical and subcommissural organs (see review Dinan and Scott, 2005). It is thought that the AVP levels in the pituitary portal vascular system is derived from the PVN, but morphological and neurochemical studies suggest that AVP from supraoptic magnocellular AVP-secreting cells also accesses the hypophyseal portal blood (Antoni et al., 1990; Dinan and Scott, 2005). AVP neurons in the medial amygdala and the bed nucleus of the stria terminalis project to the lateral septum and ventral hippocampal sites affecting memory and behavior (Caff et al., 1987; Aguilera, 2011). Finally, AVP is expressed in the suprachiasmatic nucleus (SCN), which is involved in the regulation of circadian rhythms (Rhodes and Rubin, 1999; Arima et al., 2002; Kalsbeek et al., 2010; Kondratova and Kondratov, 2012).

#### AVP receptors

The actions of AVP are mediated through interaction with specific plasma membrane receptors of target cells that have been identified and cloned (Jard et al., 1987; Birnbaumer, 2000; Derick et al., 2004). AVP receptors belong to the family of GPCRs, i.e., they transmit a signal to the cell through G-protein (guanyl-nucleotide-binding protein) (Jard et al., 1987; Birnbaumer, 2000; Thibonnier et al., 2002; Derick et al., 2004). AVP receptors have been divided into three major types: V1a, V1b (or V3), and V2 according to their pharmacological and G-protein-coupled properties. The V1a and V1b subtypes are both coupled to Gq and signal via phospholipase C (Jard et al., 1987; Thibonnier et al., 2002; Roper et al., 2011). The V2 receptor subtype is coupled to Gs that signal via adenylate cyclase (Thibonnier et al., 2002; Derick et al., 2004). The V1a receptor is predominantly found in vascular smooth muscle where it is involved in control of vasoconstrictor effects and blood pressure (Koshimizu et al., 2006; Vantyghem et al., 2011). The renal V2 AVP receptor is responsible for water resorption in collecting ducts of the kidney by promoting the translocation of aquaporin-2 channels to the plasma membrane (Knepper, 1997). The V1b receptor is primarily located in corticotrophs of the anterior pituitary where it stimulates ACTH release (Antoni, 1993; Derick et al., 2004; Roper et al., 2011). A number of studies utilizing *in situ* hybridization histochemistry and reverse transcription-polymerase chain reaction in rodents have shown that V1b receptor mRNA is also expressed at a lower level in some brain regions (hippocampus, PVN, olfactory bulb, and amygdala) and a number of peripheral tissues (kidney, pancreas, heart, lung, breast, adrenal medulla, and others) (Grazzini et al., 1996; Roper et al., 2011), where its function is unknown (Roper et al., 2011).

#### Effects of AVP on the HPA axis function and interaction with CRH

The ability of both neuropeptides CRH and AVP to stimulate the secretion of ACTH has been demonstrated in humans (DeBold et al., 1984; Salata et al., 1988; Favrod-Coune et al., 1993) and various other species, including non-human primates (Goncharova and Lapin, 2002; Goncharova, 2009a), rats (Vale et al., 1983; Antoni, 1993), mice (Muller et al., 2000; Lolait et al., 2007), horses (Evans et al., 1993), and sheep (McFarlane et al., 1995; Hassan et al., 2003). The relative importance of each seems to vary depending on the species. For example, in humans (DeBold et al., 1984; Favrod-Coune et al., 1993), non-human primates (Goncharova, 2009a), rats (Vale et al., 1983; Antoni, 1993; Serradeil-Le Gal et al., 2005), and horses (Evans et al., 1993), CRH is a much more potent secretagogue than AVP. At the same time, some groups have reported that in the sheep AVP is the more potent secretagogue (Familari et al., 1989; Hassan et al., 2003). In all cases, a marked synergism between CRH and AVP has been observed (Antoni, 1993; Favrod-Coune et al., 1993; Aguilera et al., 1994; Aguilera, 1998; Serradeil-Le Gal et al., 2005). The impact of AVP on ACTH secretion is often regarded as ancillary to CRH as far as AVP alone is a weak ACTH secretagogue but acts synergistically with CRH to facilitate ACTH release both in humans (DeBold et al., 1984; Salata et al., 1988; Favrod-Coune et al., 1993) and rodents (Rivier and Vale, 1983; Antoni, 1993). It has been shown that the corticotroph AVP/V1b and the CRH/CRHR1 signaling pathways converge to increase ACTH secretion (Abou-Samra et al., 1986; Roper et al., 2011). In particular, it has been suggested that AVP plays a role in stimulating the primary nuclear transcripts induced by CRH in pituitary corticotrophs (Antoni, 1993). Studies in recent years suggest that the CRH–AVP synergism may also be conditioned by change in the physical state of corticotroph V1b and CRHR1 receptors (Young et al., 2007; Roper et al., 2011). The two receptors may physically heterodimerize (Young et al., 2007).

## Vasopressinergic Regulation of Stress Responsiveness of the HPA Axis

### AVP in regulation of HPA axis reactivity in response to acute stress

#### Acute stress

Acute immobilization stress has been observed to lead to up-regulation of AVP mRNA along with up-regulation of CRH mRNA expression in parvicellular neurons of the PVN (Bartanusz et al., 1993). Moreover, compensatory increase in basal vasopressinergic system in mice lacking the CRHR1 gene was revealed (Muller et al., 2000). All this allows us to consider AVP as an important modulator of the ACTH response to stress, as a compensatory mechanism to maintain the activity of the HPA axis when the CRH/CRHR1 signaling pathway is damaged (Muller et al., 2000). The importance of AVP along with CRH as physiological modulators of ACTH secretion in response to insulin-induced hypoglycemia has been demonstrated in humans (DeBold et al., 1984; Ellis et al., 1990).

The precise role of AVP production in response to stress remains controversial because most studies have involved indirect or correlative measurements of activation of AVP-producing neurons after exposure to stress (DeBold et al., 1984; Ellis et al., 1990; Makara et al., 2004; Lolait et al., 2007). Some of the best evidence for AVP involvement in the response of the HPA axis response to stress is derived from studies that have shown that immunoneutralization of AVP diminish the increase in plasma ACTH produced by various stressors such as restraint, insulin-induced hypoglycemia, or injection of bacterial lipopolysaccharide (LPS) (Turnbull et al., 1998; see introduction in Lolait et al., 2007). In addition, studies in which AVP levels have been sampled in pituitary portal blood indicate that AVP may be secreted preferentially over CRH in response to acute stressors such as insulin-induced hypoglycemia (Plotsky et al., 1985; Engler et al., 1989).

Nevertheless, several studies do not support the conclusion of an important role for AVP in the regulation of HPA axis stress responsiveness. Animals in some studies using modern genetic and pharmacological models to create an AVP deficit have, nevertheless shown a normal response of the HPA axis in acute stress, specifically to restraint, resident-intruder, and LPS stressors (Lolait et al., 2007; Chen et al., 2008a; Zelena et al., 2011). For example, mice lacking AVP V1b receptors exhibited normal corticosterone responses to acute physical–psychological stress in the resident-intruder paradigm (Wersinger et al., 2002).

In other studies using genetic and pharmacological models of AVP deficit the pituitary–adrenal responses to acute stress were substantially reduced. Thus, recent studies with the V1b receptor knockout mice demonstrated reduced plasma levels of ACTH and corticosterone or only ACTH in response to insulin-induced hypoglycemia (Lolait et al., 2007), forced-swimming (Tanoue et al., 2004; Stewart et al., 2008), novel environment (Stewart et al., 2008), mild restraint (Stewart et al., 2008), and in response to some of the other stressors (Roper et al., 2011) compared to wild-type mice. The use of SSR149415, the first selective V1b receptor antagonist, at 2 h after treatment significantly suppressed the increase in ACTH after a 5 min forced-swimming stress and decreased the dramatic elevation in plasma corticosterone concentrations in mice (Serradeil-Le Gal et al., 2005). Acute administration of another antagonist of V1b receptor (Org) also reduced ACTH secretion following both restraint and LPS but did not antagonize the ACTH response to noise (Spiga et al., 2009). Interestingly, the Org V1b antagonist, while reducing the stress-induced increase in ACTH secretion, had no effect on plasma corticosterone levels (Spiga et al., 2009). Similar results were obtained when another V1b receptor antagonist (SSR149415) was used: the ACTH plasma level was reduced in rats in response to restraint compared to controls, but showed no inhibitory effect on the secretion of corticosterone (Serradeil-Le Gal et al., 2003).

Findings from studies of the role of AVP in responsiveness of the HPA axis in acute stress using V1b receptor antagonists in rodents are consistent with studies of naturally AVP-deficient Brattleboro rats (Zelena et al., 2009; Makara et al., 2012). The ACTH response of homozygous animals to severe stressors, such as those mentioned above, as well as to anaphylactoid stress (hypertonic saline, egg white injection), elevated plus-maze, and novelty, was sharply decreased compared to that of heterozygous littermates. The ACTH response also was decreased in response to volume load, restraint, or aggressive attack in AVP-deficient rats. At the same time, there were no significant differences in the ACTH reaction in response to such stressors as social avoidance, footshock, and ether inhalation. Differences were also observed in the corticosterone response to the three groups of stressors. It did not change in response to the first group of stressors, decreased in parallel with ACTH in AVP-deficient rats in response to the second group of stressors and, like ACTH, did not change, in response to the third group of stressors (Zelena et al., 2009; Makara et al., 2012). Herman et al. (1996) and Zelena et al. (2009) suggest that the brain categorizes stressors and utilizes neural response pathways that vary in accord with their assigned category as well as that AVP increase the ACTH response to stress in a context-specific manner (Zelena et al., 2009).

Age can also influence the importance of AVP for the HPA axis response to acute stress. Reduced ACTH and corticosterone release was observed in AVP-deficient rats in the neonatal period, while adult rats of the same strain demonstrated a normal response of the pituitary–adrenal axis to LPS injection (Zelena et al., 2011).

#### Basal conditions

There is a wide range of studies on the role of AVP in the HPA axis regulation not only in response to acute stressors but during basal conditions. Studies using AVP immunoneutralization have not identified a role of AVP in basal ACTH regulation (Ono et al., 1985; Tilders et al., 1985). Studies using genetic models of AVP deficit based on knocking out the corticotroph V1b receptor resulted either in reduced (Tanoue et al., 2004) or normal (Lolait et al., 2007) resting ACTH and corticosterone levels. A selective V1b receptor antagonist (Org), which decreased restraint- and LPS-induced ACTH secretion, did not affect the resting levels or stress-induced corticosterone responses (Spiga et al., 2009). The resting pituitary ACTH concentration of homozygous Brattleboro rats appeared to be normal (Lolait et al., 1986; Zelena et al., 2009), although there was a trend toward elevated corticosterone at decapitation (Zelena et al., 2009) and also there are publications of impaired basal ACTH and corticosterone plasma levels (Burgess and Balment, 1992).

### AVP regulation of HPA axis responsivity to chronic stress

Regulation of V1b receptors in the pituitary gland appears to play a major role in corticotroph responses to chronic stressors, as a good correlation has been established between the concentration of the receptor and the secretion of ACTH by the pituitary gland (Aguilera, 1994; Aguilera et al., 1994). Furthermore, increased expression of AVP mRNA in parvicellular neurons of the PVN has been demonstrated along with a positive correlation between the density of corticotroph V1b receptors and ACTH release in response to chronic stress (Aguilera, 1994).

Numerous studies performed in rats have revealed a shift of hypothalamic CRH/AVP signal in favor of AVP in some chronic stress states. This is manifested by enhanced AVP synthesis in CRH-producing cells in parvicellular neurons of the PVN after repeated restraint (Whitnall, 1989; De Goeij et al., 1992; Bartanusz et al., 1993; Aguilera, 1994), foot shock (Sawchenko et al., 1993), intraperitoneal injection of hypertonic saline (Ma and Aguilera, 1999), and AVP storage in the CRH-containing nerve terminals in the external zone of the median eminence (De Goeij et al., 1992). An increased proportion of hypothalamic neurons coexpressing AVP has also been demonstrated with such stressors (De Goeij et al., 1992). At the same time, CRH production remained unchanged or decreased (De Goeij et al., 1992). CRH expression increased only in cases of chronic stress when circulating ACTH and glucocorticoid levels increased, for example, in response to stress caused by repeated injections of intraperitoneal hypertonic saline or footshock (Imaki et al., 1991; Ma and Aguilera, 1999).

The results of direct manipulation of the HPA axis reinforce the idea that chronic stress induces a shift of the hypothalamic CRH/AVP signal in favor of AVP. Prolonged administration of CRH by osmotic minipumps led to a reduction in CRH receptor number in the pituitary corticotrophs of control rats (Tizabi and Aguilera, 1992), which was enhanced by the simultaneous infusion of AVP. At the same time adrenalectomized Brattleboro rats lacking hypothalamic AVP, showed only a minimal reduction in CRH receptor number, which was increased by AVP infusion in the period after adrenalectomy (Tizabi and Aguilera, 1992).

Repeated restraint stress or repeated hypertonic saline injections were found to produce sustained increases in the expression of V1b receptor mRNA in the pituitary (Rabadan-Diehl et al., 1995). This suggests an up-regulation of AVP receptors in situations of chronic stress, which may explain the preserved enhanced response to novel superimposed acute stress exposure (so-called heterotypic stressor) in these animal models. Thus, the study using V1b receptor knockout mice demonstrated the importance of AVP and V1b receptor for a normal response of the HPA axis to heterotypic stressor (hypoglycemia) in chronic stress induced by repeated restraint (Tanoue et al., 2004; Lolait et al., 2007). In addition, the plasma ACTH response to repeated mild restraint, forced-swimming, and novel environments in V1b receptor knockout mice was decreased in comparison with wild-type mice (Stewart et al., 2008; Roper et al., 2011).

The sensitivity of CRH and AVP transcription to glucocorticoid feedback is markedly different (Bilezijian et al., 1987). CRH mRNA and CRH receptor mRNA levels are reduced by elevated glucocorticoids, whereas V1b receptor mRNA levels and coupling of the receptor to phospholipase C are stimulated by glucocorticoids, effects which may contribute to the refractoriness of AVP-stimulated ACTH secretion to glucocorticoid feedback (Rabadan-Diehl and Aguilera, 1998; Aguilera and Rabadan-Diehl, 2000). This suggest that vasopressinergic regulation of the HPA axis is critical for sustained corticotroph responsiveness in the presence of high circulating glucocorticoid levels during chronic stress (Rabadan-Diehl and Aguilera, 1998; Aguilera and Rabadan-Diehl, 2000).

All these findings support the proposal that CRH plays a predominantly permissive role in the HPA axis regulation in conditions of chronic stress, but AVP is a dynamic modulator of ACTH release (Plotsky, 1991; Dinan and Scott, 2005).

Some recent studies of chronic stress in naturally AVP-deficient Brattleboro rats, suggest that the role of V1b and AVP in adaptation of the HPA axis to chronic stress may not be as convincing as first thought. Experiments on Brattleboro rats and control heterozygous littermates utilized three different chronic stress models: repeated restraint to produce physical–psychological stress (Zelena et al., 2004; Makara et al., 2012); repeated short periods of morphine withdrawal (Domokos et al., 2008; Makara et al., 2012); streptozotocin induced diabetes (Zelena et al., 2006; Makara et al., 2012). The changes characteristic to chronic stress, including body weight reduction, involution of the thymus, adrenal gland hypertrophy, and increases in basal POMC mRNA and plasma corticosterone, developed in all three models, but no difference was observed between the genotypes. Both male AVP-deficient Brattleboro rats and their heterozygous littermates exposed to chronic unpredictable stress, exhibited elevated levels of POMC mRNA in the anterior lobe of the pituitary and plasma ACTH along with a less significant increase in plasma corticosterone (Varga et al., 2011). In addition, the ACTH and corticosterone response to repeated shaker stress in V1b receptor knockout mice is not different from that seen in wild-type mice (Roper et al., 2011). There are also studies using pharmacological models of AVP-deficiency that have shown adequate HPA axis responses to chronic stress in the absence of AVP (Chen et al., 2008b). These data suggest that AVP is not so important for the development of the chronic stress response.

Perhaps the contradictory results can be explained in terms of age and species differences in the HPA function of rodents and primates, including humans. Some evidence suggests that AVP is the main regulator of the HPA axis during the perinatal period (Zelena et al., 2008; Makara et al., 2012). And in some key aspects of HPA function non-human primates are a more appropriate animal model for human than rodents. For example, the adrenal cortex of monkeys, in contrast to rodents, produces DHEA and DHEAS (Goncharova, 1997; Kemnitz et al., 2000; Conley et al., 2004), which are involved in stress response of the HPA axis and may regulate the release of AVP (Deuster et al., 2005). It is apparent that we need more in-depth research on the role of AVP in the HPA axis regulation in response to different types of chronic stress on various rodent models, including both wild-type and homozygous AVP-deficient Brattleboro mutant strains, as well as V1b receptor knockout rodents. It should also investigate in detail the function of the HPA axis in AVP-deficient lines in basal conditions using various functional tests to get a solid baseline data that are comprehensively characterize the artificial models. Extensive basic research is required on the effects of V1b receptors antagonists and agonists on the HPA axis function in resting and stress conditions in primates.

## Developmental Specialties of Vasopressinergic HPA Axis Regulation

### Fetal development

Extensive clinical and experimental data indicate variation in the importance of AVP in regulation of the HPA axis, including its stress responsiveness, at different stages of ontogeny and under different physiological conditions. The role of AVP in regulation of the HPA axis in the fetus has been described only in a few studies (Carey et al., 2007, 2009). In fetal sheep late in gestation, secretion of ACTH by the pituitary gland increases in response to stimulation with AVP. This effect is associated with an increase in the generation of inositol 1,4,5-trisphosphate, a second messenger of activated V1b receptors (Carey et al., 2007). The same study also found that hypothalamo-pituitary disconnection significantly impaired this ontogenetic development and prevents the developmental increase in fetal plasma cortisol concentrations in late gestation (Carey et al., 2007). Another study confirmed the hypothesis that cortisol is an important modulator of pituitary responsiveness to AVP in the late gestation sheep fetus (Carey et al., 2009).

### Neonatal period

Developmental studies have shown that neonatal rodents (Yi and Baram, 1994; Dent et al., 1999; Schmidt et al., 2009; Zelena et al., 2011) and humans (Gunnar and Donzella, 2002) show a reduced adrenocortical response to stress. Thus, this period is called the stress-hyporesponsive period (Yi and Baram, 1994; Dent et al., 1999; Schmidt et al., 2009). In some studies the reduced response of the pituitary–adrenal axis to stressors was not associated with significant change in CRH gene transcription (Yi and Baram, 1994) or even mentioned its decrease in response to certain stressors, such as endotoxin (Dent et al., 1999). The use of conditional knockout mice with a deletion of the GR at the pituitary (GRPOMCCre) showed that during the neonatal period expression of CRH mRNA in parvicellular neurons of the PVN is reduced while expression of AVP mRNA is increased (Schmidt et al., 2009). Similar data were obtained in 10-day-old pups of AVP-deficient Brattleboro rats, which showed significantly lower resting CRH mRNA levels in the PVN and have revealed that AVP-deficiency abolishes the ACTH stress response in perinatal rats compared to control littermates (Zelena et al., 2008). Recent studies by this research group using the V1b antagonist, SSR149415, confirmed a primary role for AVP in ACTH stress responsiveness of control rats during the neonatal period (Zelena et al., 2011; Makara et al., 2012). Apparently, the shift in the ratio CRH/AVP in favor AVP (Whitnall, 1989; Dinan and Scott, 2005) can take place not only in adult individuals under chronic stress or acute stress induced by a severe stressor, as noted in Section “Vasopressinergic Regulation of Stress Responsiveness of the HPA Axis” above but also in the neonatal period.

### Late pregnancy, lactation

Such important physiological conditions of the organism as pregnancy and lactation are of considerable interest in terms of vasopressinergic regulation of stress reactivity of the HPA axis. It is well known that the HPA axis response to stressors is markedly attenuated in late pregnancy and during lactation period in women (Altemus et al., 1995; de Weerth and Buitelaar, 2005) and female rodents (Fischer et al., 1995; Neumann et al., 1998; Walker et al., 2001; Ma et al., 2005; Brunton et al., 2008). The precise mechanisms of maternal HPA axis hyporesponsiveness in these two states are different (see review Brunton et al., 2008), but both states are characterized by marked changes in AVP secretion from parvicellular neurons of the PVN and subsequent change in the secretion of ACTH in response to stress. The rat’s response to stress exposure late in pregnancy is associated with reduced AVP expression (Ma et al., 2005) or reduced expression of both AVP and CRH (da Costa et al., 1996; Douglas et al., 2003). On the contrary, AVP mRNA expression in parvicellular neurons of the PVN in postpartum females exposed to restraint stress during lactation was greater than that of virgin females (Fischer et al., 1995; Walker et al., 2001). At the same time, the expression of CRH mRNA in the PVN significantly decreased during lactation (Fischer et al., 1995; Shanks et al., 1999; Walker et al., 2001). It suggests that AVP may play an important role in the stimulation of ACTH secretion during lactation in rats (Walker et al., 2001). Thus, as in the fetus and neonate, AVP performs an important role in regulation of the HPA axis stress reactivity with a shift in the CRH/AVP ratio in favor of AVP in pregnant and lactating females.

Gestation and early postnatal periods, as we know, are the most vulnerable periods of ontogenesis in terms of programing disruptions of the HPA axis for adulthood (Schmidt et al., 2009; Long et al., 2010; Renard et al., 2010) and aging (Goncharova, 2009b; Solas et al., 2010). Reduced HPA axis response to stressors during late pregnancy, lactation, and the neonatal period, apparently, is an adaptive response of the body that provides protection of the fetus and newborn from an excess of toxic glucocorticoids and that can also have a programing effect on the development of the HPA axis and other physiological systems in later life.

## AVP in Regulation of HPA Axis Stress Responsiveness during Aging

### Activity of the HPA axis during aging

It is generally accepted that mainly hyperactivation of the HPA axis occurs during aging. In small laboratory animals hyperactivation of the HPA axis in the aging process develops at all levels of the system. Thus, aged rodents demonstrated elevated resting ACTH and corticosterone plasma levels (Sapolsky et al., 1986; Tizabi et al., 1992; Hauger et al., 1994; Sapolsky, 1999; Meijer et al., 2005; Lo et al., 2006), an increased release of resting hypothalamic CRH (Scaccianoce et al., 1990; Hauger et al., 1994; Sapolsky, 1999), a decreased sensitivity of the adrenal glands to ACTH (Tang and Phillips, 1978; Sapolsky et al., 1986), of the anterior pituitary to CRH (Hylka et al., 1984; Sapolsky et al., 1986; Hauger et al., 1994; Sapolsky, 1999), of the pituitary, hippocampus, and hypothalamus to the level of circulating glucocorticoids (Sapolsky et al., 1986; Tizabi et al., 1992; Hatzinger et al., 2000; Revskoy and Redei, 2000). In old rodents, hyperactivity of the HPA axis is likely to reflect loss of resiliency and reduced sensitivity to negative glucocorticoid feedback, which mainly reflects hippocampal receptor damage (Sapolsky et al., 1986; Seeman and Robbins, 1994; Pedersen et al., 2001; Giordano et al., 2005). As a result, older animals tend to show a more prolonged elevation of corticosterone in response to stress exposure (Sapolsky et al., 1986; Seeman and Robbins, 1994; Sapolsky, 1999). Marked disturbances have been shown in the reaction of aged rat adrenals to stress.

These have been expressed mainly as increases in the maximum peak values of plasma corticosterone rise and the prolongation of elevated corticosterone secretion (Sapolsky et al., 1986; Seeman and Robbins, 1994; Sapolsky, 1999; Lo et al., 2006). Some studies, however, have shown individual and species differences ranging from unchanged and reduced basal and stress responses in aged rats compared to young rats (Cizza et al., 1994; Hauger et al., 1994; Lightman et al., 2000).

Hyperactivation of the HPA axis during aging in humans and non-human primates is generally associated with elevated plasma levels of ACTH and cortisol in basal conditions (Sapolsky and Altman, 1991; Guazzo et al., 1996; Lupien et al., 1999, 2007). Decrease in HPA axis sensitivity to glucocorticoid regulation by a feedback mechanism with age has also been observed in humans (Born et al., 1995; Heuser et al., 2000; Giordano et al., 2005) and non-human primates (Sapolsky and Altman, 1991; Brooke et al., 1994; Goncharova et al., 2000; Gust et al., 2000; Goncharova and Lapin, 2002, 2004). However, in contrast to rodents there was an increase in response of the pituitary–adrenal axis to CRH and ACTH injection in humans (Born et al., 1995; Kudielka et al., 1999; Ferrari et al., 2001) and monkeys (Goncharova and Lapin, 2002, 2004; Goncharova et al., 2002), as was a higher responsiveness of the pituitary–adrenal axis to combined administration of CRH and AVP (Born et al., 1995). With healthy aging basal activity of CRH-producing neurons in the hypothalamic PVN slightly increased in the human (see review Swaab et al., 2005).

It should be noted that aging is associated with an increase in the variability of disturbances in the HPA axis both in primates and rodents but to a greater extent in humans and non-human primates than in rodents. Many authors have failed to observe significant age-related changes in basal plasma levels of ACTH and cortisol in humans (Ohashi et al., 1986; Waltman et al., 1991; Goncharova, 1997; Goncharova et al., 2002) and monkeys (Moore et al., 1979; Goncharova and Lapin, 2000, 2002; Goncharova et al., 2000; Kemnitz et al., 2000). Some even have observed decreased basal cortisol levels in humans (Blichert-Toft, 1978; Sherman et al., 1985) and monkeys (Gust et al., 2000). Other researchers have found no marked age-related changes in sensitivity of the HPA axis to inhibition by glucocorticoid feedback (Blichert-Toft, 1978; Waltman et al., 1991; Huizenga et al., 1998). There was no change in the reaction of the adrenal cortex to injection of CRH, ACTH, or insulin in humans (Blichert-Toft, 1978; Ohashi et al., 1986) or non-human primates (Goncharova and Lapin, 2000; Goncharova et al., 2000).

Some researchers have found higher responses to acute psycho-emotional stress in physically untrained aged individuals (Traustadóttir et al., 2005). Older men responded to psychological stress with increased cortisol levels and more intense cardiovascular responses compared to women of similar age (Traustadottir et al., 2003). Comparative analysis of cortisol response to various pharmacological stimuli including dexamethasone, CRH, naloxone, ACTH, insulin, epinephrine, metapyrone, physostigmine, and hypertonic saline, as well as to psychological stressors in healthy volunteers, young and old age through the analysis of the data published by different authors in 45 articles, allowed the authors (Otte et al., 2005) to come to a conclusion about a higher HPA axis response in older subjects compared to younger persons. The effect of age on the release of cortisol was significantly greater in women.

At the same time, no age-related differences in ACTH and cortisol levels in response to acute psychosocial stress have been reported in postmenopausal women compared to young women (Kudielka et al., 1999). The response to acute psychological stress in the elderly who were aerobically fit did not differ from that of young adults (Traustadóttir et al., 2005). Marked difference in function of the HPA axis in response to surgical stress is not seen between young persons and centenarians (Kudoh et al., 2001), even though clinical observations indicate that the mortality rate of elderly patients, undergoing surgery is much higher than that of young patients (Blichert-Toft, 1978). The higher rate may be due to reduced adaptive ability of the aging body and deterioration of HPA axis function.

Experiments on healthy non-human primates (*Macaca mulatta*) revealed that age-related dysfunctions of the HPA axis are associated with adaptive behavior of animals (Goncharova et al., 2010). Monkeys with depression-like behavior show age-related changes in the HPA axis function that are accompanied by maximal absolute and relative hypercortisolemia (high cortisol/DHEAS molar ratio) under resting conditions, as well as by a significantly greater increase in plasma cortisol levels with acute psycho-emotional stress. Young aggressive monkeys, in comparison with young monkeys of other behavior groups, demonstrated the highest plasma levels of DHEAS and the lowest molar ratios of cortisol to DHEAS. Such differences were not exhibited by old monkeys with aggressive behavior. Minimal age-related changes in the HPA axis have been observed in monkeys with average (standard) behavior.

It should be noted that a striking difference in physiology of the HPA axis between primates and rodents is the secretion of large amounts of DHEA and DHEAS by the primate adrenal cortex, which undergoes a decline with age (Orentreich et al., 1992; Goncharova, 1997; Yen and Laughlin, 1998; Ferrari et al., 2001; Goncharova and Lapin, 2002; Conley et al., 2004; Heaney et al., 2012). DHEA and DHEAS serve as precursors to androgens and estrogens (Yen and Laughlin, 1998; Labrie et al., 2001; Mellon and Vaudry, 2001). In addition, they may act as functional antagonists to the effects of glucocorticoids (Yen and Laughlin, 1998; Maninger et al., 2009). In the brain, they are neuromodulators of neuronal receptors, such as GABA-A and *N*-methyl-d-aspartate (NMDA) receptors (Baulieu, 1998; Yen and Laughlin, 1998; Mellon and Vaudry, 2001), which are part of the neurons of afferents nerves regulating – among others – the PVN activity (Arvat et al., 2002; Deuster et al., 2005).

### Circadian rhythms

Significant variability has been found with respect to age-related changes in circadian rhythms of ACTH and glucocorticoid secretion both in humans and animals. While a number of studies have failed to show marked circadian disorders of cortisol and ACTH in humans and non-human primates (Chambers et al., 1982; Waltman et al., 1991; Ceresini et al., 2000) and some strains of rats (Honma et al., 1996), an increasing number of studies have shown disruption of circadian rhythms in HPA axis activity with aging in both humans and animals. Many authors have reported age-associated increases in basal ACTH and cortisol concentrations in terms of basal evening levels and nadirs in humans (Ferrari et al., 2001; Giordano et al., 2005; Hofman and Swaab, 2006) and non-human primates (Gust et al., 2000; Khavinson et al., 2001; Goncharova et al., 2002, 2006b; Zhdanova et al., 2011), and fall of the levels of ACTH and corticosterone in the evening and increase during the circadian trough in rodents (Hauger et al., 1994; Lightman et al., 2000; Froy, 2011). A damaged daily rhythm may involve the SCN, as fetal transplants containing SCN can restore the circadian rhythm in old Sprague-Dawley rats (Cai et al., 1997).

The master circadian clock is located in the SCN of the hypothalamus. Its activity is synchronized with the natural day–night cycle, and it coordinates the circadian rhythms of the body, including the HPA axis rhythm (Reppert and Weaver, 2002; Kalsbeek et al., 2010; Walker et al., 2010; Kondratova and Kondratov, 2012). A characteristic feature of the SCN in many species, including humans and other primates is a subpopulation of AVP-containing neurons (Kalsbeek et al., 2010). The SCN regulates HPA axis activity through the PVN via AVP, which serves as a neurotransmitter in the SCN afferents to the hypothalamic PVN (Kalsbeek et al., 2010). The regulatory function of these afferents is confirmed by experiments with microinjections of AVP-cytotoxic monoclonal antibodies into the SCN, which produce a significant reduction of immunoreactive AVP and AVP mRNA expression there as well as in parvicellular neurons of the PVN (Gomez et al., 1997). Study of post-mortem human brains revealed a pronounced circadian variation in the activity of AVP-containing neurons in the SCN (Kalsbeek et al., 2010). Animal experiments have shown an important role for SCN-derived AVP in the control of neuroendocrine day/night rhythms of the HPA axis (Kalsbeek et al., 2010). The number of AVP-expressing neurons in the SCN declines with age in humans (Swaab et al., 1985), non-human primates (Cayetanot et al., 2005), and rats (Roozendaal et al., 1987). Decreased AVP-producing neurons in the SCN in aged humans and animals are accompanied by deterioration of the circadian rhythms of HPA axis function (Kalsbeek et al., 2010).

### AVP regulation of stress responsiveness of the HPA axis with aging

Age-related changes in AVP production vary depending on location in the brain. While secretion of AVP decreases with aging in the SCN of animals and humans (Cai et al., 1997; Cayetanot et al., 2005; Hofman and Swaab, 2006; Kalsbeek et al., 2010), its secretion in the PVN seems to increase. This is indicated by immunocytochemical studies of human post-mortem brains that have revealed increased amounts of AVP in AVP-expressing cells and in the size of AVP-containing neurons in the PVN of aged humans (Lucassen et al., 1993; Prelevic and Jacobs, 1997; Ishunina and Swaab, 1999). In addition, an age-dependent change in colocalization of CRH and AVP in favor of AVP in the human PVN has been identified (Raadsheer et al., 1993). Moreover, old rats showed signs of hyperactive AVP-expressing neurons (Terwell et al., 1992; Bazhanova et al., 2000). Other studies, however, provide indications of a modest activation of CRH neurons with aging in healthy persons (see review Swaab et al., 2005).

In the literature there are several studies that investigated the role of the AVP/V1b receptor system in the regulation of stress responsiveness of the HPA axis with aging. Most of these studies have been performed in experiments on rodents. Features of vasopressinergic regulation of stress responsiveness of the HPA axis during aging has been studied significantly less in humans and non-human primates.

#### Rodents

On the other hand, the idea that secretion of AVP in the PVN can increase with aging is evidenced by the results of studies on the role of AVP in the regulation of stress reactivity of the HPA axis, performed mainly on rodents. Thus, some reports in the literature describe work with rats of the Fischer-344/N strain, in which aging is associated with a progressive decline in hypothalamic CRH production associated with increased production of AVP (Cizza et al., 1994). In this case, basal plasma ACTH levels were similar across age groups. Injection of exogenous rat CRH elicited significantly greater ACTH and corticosterone responses in aged rats, which was consistent with the observation of hypothalamic CRH deficiency. CRH mRNA levels in the PVN, CRH content, and *in vitro* secretion of CRH by whole explanted hypothalamus showed progressive and significant reductions with age, whereas the steady state levels of AVP mRNA significantly increased with age (Cizza et al., 1994). Perhaps the importance of PVN-derived AVP for HPA axis regulation increases with advancing age, while the importance of CRH is reduced.

Subsequent studies have confirmed the important role of AVP expressed in parvicellular neurons of the PVN in the regulation of ACTH secretion in response to stimulus. Application of the combined DEX/CRH test to old male Wistar rats resulted in excessive release of ACTH and corticosterone as compared to young rats. Administration of a V1b receptor antagonist between the dexamethasone and CRH injections blocked the effect (Hatzinger et al., 2000). In DEX-pretreated aged rats the authors detected increased numbers of AVP mRNA-expressing neurons in the PVN; this was in combination with increased numbers and activity of CRH mRNA-expressing neurons. This study provided the first direct evidence of the involvement of excessive activity of AVP-expressing neurons in age-dependent hyperactivity of the HPA axis. The results of this study suggest an important role of AVP in the mechanism of increased HPA axis activity in aging rats, possibly secondary to defective function of corticosteroid receptors (Hatzinger et al., 2000).

Another study has demonstrated an increase in the content of AVP within the PVN in basal conditions. The approximately twofold increase correlated with an increase of ACTH and corticosterone basal plasma levels in old rats (Keck et al., 2000). However, in response to acute stressor, forced-swimming, the responses of AVP, ACTH, and cortisol were significantly lower than in young animals (Keck et al., 2000). Some authors report that aging in rats is likely to be associated with reduced production of AVP and hypersecretion of CRH in the hypothalamus and subsequent down-regulation of corticotroph CRH receptor (Hauger et al., 1994).

Some of the contradictory results reported in the above papers may by attributable to individual differences in HPA axis function both in resting and stress conditions. Thus, in the study of Meijer et al. (2005) young and aged rats had previously been classified as inferior and superior learners on the basis of their performance in a water maze test. The authors tested for a correlation between the production of the hypothalamic neuropeptides, CRH and AVP, and their status as inferior and superior learners. They found that AVP expression in parvicellular neurons of the PVN was virtually unchanged with aging in animals of either group. CRH expression, however, decreased significantly in old animals compared to young animals in the superior learning group, which produced a change in the ratio of CRH/AVP expression in favor of AVP. By contrast, the ratio of CRH/AVP expression in inferior learners was virtually unchanged with aging. Apparently, there are marked individual differences in rats of the same strain, as well as differences between strains in aging of the brain in general and aging of the HPA axis in particular. This conclusion is supported by the results of studies that demonstrate differences in the functioning of the HPA axis in adult rats of the same species with high and low trait anxiety (Keck et al., 2002). Using surgical microdialysis *in vivo*, the investigators showed that the content of AVP in the PVN is significantly higher in rats with increased anxiety (Keck et al., 2002).

Thus, reports on the possible role of the AVP/V1b receptor system in the increase of HPA axis hyperactivity with aging in rodents are contradictory, suggesting (a) the possible increase of the AVP production in the PVN for aged rodents, (b) the possible lack of any age changes in the AVP secretion in basal and stress conditions, and (c) the possible reduced basal AVP production along with the CRH hypersecretion.

#### Humans and non-human primates

Only a few studies have addressed age-related changes in vasopressinergic regulation of HPA axis stress reactivity in humans and non-human primates (Raskind et al., 1995; Deuster et al., 2005; Goncharova, 2009a). Most studies have focused on age differences in response of the pituitary–adrenal axis to stress. The cross-species findings are quite variable with regard to responsiveness of the HPA axis in old age. They depend not only on the nature, intensity and duration of exposure of the stressor, the genetic characteristics of the individual, and the environment in old age, but also on the influence of genetic and environmental factors in the early stages of development, particularly the fetal and neonatal periods. Environmental stress in early life may program the future development, including disruptions of HPA axis stress responsiveness in old age (Pivina et al., 2007; Goncharova, 2009b; Solas et al., 2010).

It has been noted that the healthy elderly exhibit a more pronounced adrenocortical response to hypertonic saline infusion than young people (Raskind et al., 1995). The increase in levels of AVP and plasma osmolarity was similar in representatives of both age groups, but no rise in the levels of ACTH was observed in either older or young subjects. The authors, therefore, suggested that the increased cortisol secretion in response to stimulation with the hypertonic solution was due to age-related changes in the adrenocortical level of the HPA axis (Raskind et al., 1995). Perhaps, stimulation of cortisol secretion was influenced by increased levels of AVP in the general circulation. This effect could be a direct AVP effect on the adrenal gland as the pituitary contains predominantly V1b receptor, but the adrenal cortex has V1a receptor (Grazzini et al., 1996; Vezzosi et al., 2007; Roper et al., 2011). Thus, there is evidence that AVP potentiates cortisol secretion *in vitro* by normal human adrenal cortex and some cortisol-producing adrenocortical tumors or hyperplasia through the activation of V1a receptors (Perraudin et al., 1993, 1995).

Interestingly, chronic administration of DHEA to healthy young men significantly increased exercise-induced release of AVP along with ACTH and cortisol secretion (Deuster et al., 2005). Perhaps, the stimulating effect of DHEA on pituitary–adrenal axis stress responsiveness is due to its action as antagonist of GABA-A receptors restrained the HPA axis and the stimulating effect of DHEA on NMDA receptors, involved in AVP release (Rossi and Chen, 2002; Deuster et al., 2005). In this regard, DHEA deficiency during aging in humans and non-human primates may play an important role in development of the age-related disturbances of the HPA axis and, in particular, of its stress responsiveness (Kudielka et al., 1998; Goncharova et al., 2000).

Recent experiments on non-human primates (Goncharova, 2006, 2009a; Goncharova et al., 2006a, 2008b), had shown that stress responsiveness of the HPA axis depends to a large extent on the time of day when stimulus is applied as well as on initial sensitivity of the HPA axis. It was shown that the circadian rhythm of ACTH and cortisol level is evident not only in basal conditions but also in response to acute psycho-emotional stress. Young (6–8 years old) female rhesus monkeys showed a much higher increase in ACTH and cortisol levels in response to a 2 h restraint stress imposed at 15:00 h than to the same stress imposed at 09:00 h. This difference attenuates with aging. Old (20–27 years old) female animals showed much lower responsiveness of the HPA axis to the afternoon stress and a tendency toward higher responsiveness to the morning stress. A similar circadian rhythm in responsiveness of the HPA axis to mild acute stress was observed in young pregnant women (De Weerth et al., 2007). The time-dependent responsiveness of the HPA axis to acute immobilization stress was observed in young male *Papio hamadryas* (Chirkov et al., 1987) and young female Wistar rats (Pivina et al., 2007). Moreover, senescent rats demonstrated flattening of circadian rhythm in stress reactivity of the HPA axis (Pivina et al., 2007).

The attempt to understand the mechanism of decreasing stress reactivity of the pituitary–adrenal axis with aging led to a series of experiments with the administration of a standard dose of CRH and AVP (1 μg/kg b.w., intravenously) to young and old female rhesus monkeys at different times of day (09:00 and 15:00 h). The response of the pituitary–adrenal axis to the injection of CRH revealed a circadian rhythm with a more pronounced response in the afternoon; this rhythm was not affected by aging (Goncharova, 2009a). Apparently, the circadian rhythm in the secretion of CRH underlies the circadian rhythm in the stress reactivity of the pituitary–adrenal axis of the HPA axis. At the same time, the rise of ACTH and cortisol levels in response to AVP injection did not show a dependence on the time of the day in either young or old animals. A significant age-related difference in the rise of ACTH and cortisol in response to AVP injection was, however, found in the response to AVP administered in the afternoon; lower values were seen in older animals (Goncharova, 2009a). It appears that age differences in response of the anterior pituitary to AVP may underlie age-related differences in stress responsiveness of the HPA axis in primates in the afternoon. Perhaps age differences in response of the anterior pituitary to AVP underlie age-related differences in stress responsiveness of the HPA axis in primates in the afternoon.

## Conclusion

(1) The role of CRH and AVP in the stimulation of ACTH secretion varies depending upon the species. For humans, non-human primates, and rats CRH seems to be a more important secretagogue than AVP, in humans, all parvicellular neurons of the PVN, which produce CRH in the basal conditions, produce AVP as well, whereas in rats not more than half produce AVP.
(2) AVP is an important regulator of the ACTH response to acute stress. AVP contributes to the acute ACTH secretion to stress in a context-specific manner. AVP is needed for the acute ACTH secretion for most of the known stressors.
(3) Current data on the role of AVP in the regulation of HPA axis responsiveness to chronic stress in adulthood are contradictory and require further research.
(4) The importance of the vasopressinergic regulation of stress responsiveness of the HPA axis is elevated during the fetal development, in neonatal period, and in the lactating adult.
(5)
  (a) Aging is associated with increased variability in several parameters of HPA axis function including basal state, responsiveness to stressors, and special testing. This increased variability in the aged compared to normal adults has been observed in some form in multiple species including rodents, non-human primates, and humans.
  (b) For the most experiments on rodents, the hyperactivation of the HPA axis was observed at all levels of the HPA organization with aging under the basal and stress conditions.
  (c) With healthy aging of non-human primates and humans the HPA hyperactivation is usually associated with the increase of the cortisol level in the evening time, the slight changes in the regulation of the HPA axis by glucocorticoid feedback with relative hypercortisolemia due to the decline in secretion of the adrenal antagonists of cortisol – DHEA and DHEAS.
(6) Reports on the possible role of the AVP/V1b receptor system in the increase of HPA axis hyperactivity with aging are contradictory, suggesting
  (a) the possible increase of the AVP production in the PVN for aged rodents and humans,
  (b) the possible lack of any age changes in the AVP secretion in basal and stress conditions,
  (c) the possible reduced basal AVP production along with the CRH hypersecretion.
(7) Age-related changes in response of the HPA axis to the moderate acute restraint stress were observed in rhesus monkeys with much higher increase of ACTH and cortisol secretion in young monkeys in response to the stress imposed at 15:00 h. In addition, these age changes were associated with age-related disturbances in vasopressinergic regulation.

## Conflict of Interest Statement

The authors declare that the research was conducted in the absence of any commercial or financial relationships that could be construed as a potential conflict of interest.
